# Critical Difference and Biological Variation in Biomarkers of Oxidative Stress and Nutritional Status in Athletes

**DOI:** 10.1371/journal.pone.0149927

**Published:** 2016-03-01

**Authors:** Nathan A. Lewis, John Newell, Richard Burden, Glyn Howatson, Charles R. Pedlar

**Affiliations:** 1 English Institute of Sport, Athletes Training Village, University of Bath, Bath, United Kingdom; 2 School of Sport, Health and Applied Science, St Mary’s University, Twickenham, London, United Kingdom; 3 ORRECO, Institute of Technology, Sligo, Ireland; 4 School of Mathematics, Applied Statistics and Mathematics, National University of Ireland, Galway, Ireland; 5 Department of Sport, Exercise and Rehabilitation, Northumbria University, Newcastle, United Kingdom; 6 Water Research Group, School of Environmental Sciences and Development, Northwest University, Potchefstroom, South Africa; 7 Cardiovascular Performance Program, Massachusetts General Hospital, Boston, Massachusetts, United States of America; University of Nebraska-Lincoln, UNITED STATES

## Abstract

The longitudinal monitoring of oxidative stress (OS) in athletes may enable the identification of fatigued states and underperformance. The application of OS biomarker monitoring programs in sport are hindered by reliability and repeatability of in-the-field testing tools, the turnaround of results, and the understanding of biological variation (BV). Knowledge of BV and critical difference values (CDV) may assist with data interpretation in the individual athlete. Methods: We aimed firstly to assess the repeatability of the clinical point of care redox test, Free Oxygen Radical Test (FORT) and the Free Oxygen Radical Defence (FORD) in trained participants and elite athletes and secondly to calculate the analytical, BV, CDV and index of individuality (II) for FORT, FORD, red blood cell glutathione, lutein, α and γ–tocopherol. Part 1: Fifteen elite athletes were sampled in duplicate for calculation of the repeatability of the FORT and FORD tests. Part 2: Twelve well-trained athletes had venous samples drawn every 2 hours from 0800 to 1800 for calculation of BV, CDV, II for FORT, FORD, RBC GSH, lutein, α-tocopherol and γ–tocopherol. Results: Repeatability of the FORT and FORD assay was 3.9% and 3.7% respectively. Biomarker CDV ranged from 12.8% to 37%, with a circadian effect for FORT, α-tocopherol and γ-tocopherol (p<0.01), with all biomarker indices of individuality < 0.8 arbitrary units. Conclusion: We report that the use of the novel redox test in athletes is practical, and the generation of BV and CDV for biomarkers of OS enhances the interpretation of physiologically meaningful changes in individuals above the use of clinical reference ranges alone.

## Introduction

Exercise results in the production of reactive nitrogen and oxygen species (RNOS) and oxidative stress (OS), an effect observed after all types of exercise and consistent across species, and in different cell types, body fluids and tissues [1,2]. RNOS serve as important signaling molecules in mitochondrial biogenesis [3], glucose transport [4] and muscle hypertrophy [5,6]; thus having a role in adaptation to exercise. Indeed, exercise training increases endogenous antioxidant enzymes along with biomarkers of cell lipid and protein oxidation, with changes proportional to training volume and load [1,7,8]. The monitoring of OS in elite athletes may enable the identification of fatigued states, under performance, and illness [1]. However, the successful application of OS biomarker monitoring programs in elite sport is hindered by: 1) repeatability of in-the-field testing tools and the need for rapid turnaround of results; 2) an understanding of the athletes’ individual biological variation (BV) to elucidate meaningful changes [1,9] and 3) the inherent costs of the analytical methods and the invasive nature of repeated venous sampling. Therefore the monitoring of OS, with a point of care (POC) test, coupled with knowledge of the specific BV may assist practitioners with exercise prescription, recovery modalities and dietary interventions.

Several POC tests have been developed for ‘field use’ to measure OS [10]; although convenient, their validity has been questioned [11,12]. The Free Oxygen Radical Test (FORT) is an indirect measure of reactive intermediary by products of *in vivo* lipid, protein, and nucleic acid oxidation (hydroperoxides). The Free Oxygen Radical Defence test (FORD) is an indirect measure of antioxidant capacity, with ascorbic acid, glutathione, and albumin (but not uric acid), accounting for the majority of antioxidant activity [10]. Together, the FORT and FORD tests (FFT) provide an index of OS, and this index has been reported to have good reliability [13–15]. Clinical studies have identified significance differences for FORT values in type-2 diabetics [15], and in sickle cell disease [16] compared to healthy, age, weight and sex matched controls; diseases in which OS is a component of the pathophysiology. To date, only one case study has explored the application of the FFT in elite sport [17], in which elevated OS was demonstrated in two premier league footballers with sickle cell trait; a condition recognised for disturbed redox homeostasis and increased OS.

Practitioners, scientists and clinicians are required to decide whether changes are of physiological importance and relevant to athlete health and performance. Most commonly, interpretation of biomarker changes are made within the context of a population-based, reference-derived value [18]; such an approach has limited value because of biological variation (BV) in each biomarker. However, critical difference values (CDV), also referred to as a reference change value, can aid the interpretation of meaningful changes [9,19]. Specifically, calculation of the BV allows the quantification of random variation and temporal changes around a “set” point. For a change to be deemed physiologically meaningful in an individual, the difference in serial numerical results must be greater than the components of variation, referred to as the CDV. Furthermore, the index of individuality (II) can be used to evaluate the usefulness of population-based biomarker reference intervals for interpreting meaningful changes in individuals [18]. To date, no studies have reported the BV for the redox measures, FORT, FORD, GSH, lutein or γ-tocopherol; with recommendations made for the publication of research on the BV of biomarkers of OS in athletes [1]. Consequently, for the first time, we aimed to: 1) assess the repeatability of the FFT at rest in elite athletes, and 2) calculate the analytical variation, BV, CDV, and index of individuality for FFT, RBC GSH, lutein, and α and γ-tocopherol in well-trained participants.

## Methods

### Part 1: Repeatability

Following institutional ethical approval, 15 national and internationally ranked (includes Olympic and world medallists) endurance athletes (n = 8 males and 7 females; age (mean ± SD) 22 ± 4 y; body mass 72 ± 7.1 kg; height 1.79 ± 0.03 m; $\dot{V}$O_2max_ 66 ± 6.5 ml.kg^-1^.min^-1^) volunteered to participate. Athletes were free living and attending a national training centre, not taking any medications, and were subject to United Kingdom Anti-doping controls and testing procedures. All athletes were tested in the general preparation phase of the annual cycle and where following nutritional guidelines administered via the English Institute of Sport system. Testing was carried out between 8 a.m. and 9 a.m. in a fasted, hydrated and rested state. The following tests were performed after informed consent was obtained as a part of sports science support provision, with procedures approved by the Internal Review Board of the English Institute of Sport. Written informed consent being obtained from the athletes.

### Blood sampling

Whole blood capillary samples (50 μL for FORD, and 20μL for FORT) were taken from each ear lobe (duplicate samples), processed and analysed immediately at room temperature in line with the manufacturer’s instructions (Callegari SpA, Catellani Group, Parma, Italy). Briefly, heparinized capillary samples are immediately mixed with the reagent, centrifuged and analysed colorimetrically (CR3000, Callegari SpA, Catellani Group, Parma, Italy).

### Biochemical analysis

#### FORT assay

The presence of reactive oxygen species was determined indirectly through the FORT test [(Free Oxygen Radicals Testing) Callegari, Palma, Italy]. FORT is a colourimetric assay based on the capacity of transition metal ions (Fe^3+^ /Fe^2+^) to catalyze the breakdown of hydroperoxides (R-OOH) into derivative radicals [alkoxyl (R-O^•^) and peroxyl radicals (R-OO^•^)] within the biological sample. The application of an acidic buffer to the 20μL capillary sample, releases the transition metals from associated proteins, which react with the hydroperoxides present in the sample, producing the alkoxyl and peroxyl radicals. The derivative radicals are trapped through the addition of a buffered chromogen (reagent; an amine derivative, CrNH_2_) and develop into a radical cation in a linear based reaction at a controlled temperature of 37°C, photometrically detectable at 505nm.

$$\begin{array}{l} \text{R}-\text{OOH}+{\text{Fe}}^{2+}\to \text{R}-{\text{O}}^{•}+{\text{OH}}^{-}+{\text{Fe}}^{3+}\\ \text{R}-\text{OOH}+{\text{Fe}}^{3+}\to \text{R}-{\text{OO}}^{•}+{\text{H}}^{+}+{\text{Fe}}^{2+}\\ {\text{RO}}^{•}+{\text{ROO}}^{•}+2{\text{CrNH}}_{2}\to {\text{RO}}^{-}+{\text{ROO}}^{-}+[\text{Cr}-{\text{NH}}_{2}{}^{+•}] \end{array}$$

The intensity of the sample colour correlates with the quantity of radical compounds and therefore the concentration of hydroperoxides in the biological sample, according to Lambert-Beer’s law. The results are expressed as equivalent concentrations of H_2_O_2_ mmol•L^-1^ and linearity ranged from 1.22 to 4.56 mmol•L^-1^ H_2_O_2_.

#### FORD assay

The FORD test (Callegari, Catellani, Italy) determines the presence of plasma antioxidants via a colourimetric assay based on the capacity of the sample to reduce a preformed radical cation. In the presence of an acidic buffer and a suitable oxidant (FeCl_3_), the chromogen that contains 4-amino-N,N-diethylaniline sulfate forms a stable and coloured radical cation, photometrically detectable at 505 nm. The antioxidant compounds present in the plasma sample reduce the radical cation of the chromogen, quenching the colour, and causing a discolouration of the sample, proportional to the concentration of antioxidants present. The absorbance values generated are compared to standard curves derived from Trolox (6-hydroxy-2,5,7,8-tetramethylchroman-2-carboxylic acid), a derivative of vitamin E with enhanced water solubility. FORD values are reported as Trolox equivalents, mmol•L^-1^, linearity ranged from 0.25 to 3.0 mmol•L^-1^ Trolox.

$$\begin{array}{l} \text{Chromogen}(\text{uncolored})\;+\;\text{oxidant}\;({\text{Fe}}^{3+})\;{\text{H}}^{+}\to {\text{Chromogen}}^{.}+(\text{colour})\\ {\text{Chromogen}}^{.}+(\text{colour})\;+\;\text{AOH}\to \text{Chromogen}+(\text{uncolored})\;+\;\text{AO} \end{array}$$

### Part 2: Biological Variation:

The ethics committee of St Mary’s University approved the study. Twelve well-trained male participants (n = 12) were recruited (age (mean ± SD) 30 ± 7 y; weight 81.9 ± 8.2 kg; height 1.86 ± 0.1 m). All participants provided written, informed consent following completion of a health questionnaire. Healthy participants were selected based on recommendations for performing studies on BV [18]. Strict control of environmental factors was undertaken to reduce variability and control for factors known to disrupt redox homeostasis e.g. exercise [20], high fat meal [21], infection [22], metabolic disease [23], antioxidant supplements [4]. All participants abstained from physical exercise, alcohol consumption for 72 hours prior to testing, caffeine for 24 hours, and maintained their normal dietary habits the day before testing.

Participants arrived at the laboratory at 7.30 a.m. Venous and capillary blood samples were collected every two hours through the day at 0800, 1000, 1200, 1400, 1600, 1800 for the analysis of RBC GSH, α and γ tocopherol, lutein, and the FORT and FORD assay. To minimise sources of variation the following criteria were applied; fasted on arrival to the laboratory and remaining without food until after the last blood sample was collected at 1800 to control for hormonal fluctuations; water was allowed *ad libitum* throughout only, remaining supine on an examination couch at a comfortable temperature throughout (20–22°C) for a minimum of 20 minutes prior to each blood draw.

### Blood sampling

Blood was sampled at the antecubital vein for a total of six blood draws over 10 hours. For the RBC GSH a single 5 ml sample of venous blood was collected in a lithium heparin vacutainer tube (BD system; New Jersey, USA), and for lutein, α and γ tocopherol, a single 5 ml sample of venous blood collected in a serum separator (SST) vacutainer tube (BD system; New Jersey, USA). For details on the blood sampling of FFT see part 1 (above). For RBC GSH, lutein, α and γ tocopherol, assays the samples were centrifuged and aliquots were stored at -50°C for later analysis. To minimise analytical variance, RBC GSH, lutein, α and γ-tocopherol were analysed under the same analytical conditions; the same batch of reagents and standards, and by the same laboratory biochemists using the same analysers.

### Biochemical Analysis

Serum α-tocopherol, γ-gamma tocopherol and serum lutein were measured by reverse-phase high-pressure liquid chromatography (HPLC) on an Agilent 1200 series system (Agilent, Manchester, U.K) with ultra-violet/visible detection using a modification of the method of Thurnham *et al*[24]. Samples were protected from light on analysis to reduce oxidation, and serum separated for analysis by centrifugation at 3000 rpm for 10 minutes. Calibration was carried out using pure tocopherol standards (Sigma chemical Co, Poole, Dorset, U.K) dissolved in ethanol and a lutein standard (AASC Ltd, Southampton, U.K.) dissolved in hexane/chloroform, in which concentrations were derived by scanning ultra-violet spectrophotometry and applying the molar extinction coefficients for each substance. Quantification involved internal standardization and dose-response curves established with authentic standards. Serum α-tocopherol, γ-gamma tocopherol and serum lutein were reported as μmol•L^-1^. Intra assay CV were 3.3%, 6.8%, and 3.5% for α-tocopherol, γ-tocopherol and lutein respectively.

RBC GSH was measured by the method of Beutler *et al*. [25] using the chromogenic reaction of 5,5-dithiobis-(2-nitrobenzoic acid) (DTNB) with sulphydryl groups. The millimolar extinction coefficient of the DTNB anion was applied to derive concentrations of GSH in whole blood and the erythrocyte GSH was calculated using the haematocrit (packed cell volume) of the blood sample. RBC GSH reported as mmol GSH per litre of red cells, with the intra-assay CV for RBC GSH of 2.4%.

### Statistical Analysis

Numerical (mean ± standard deviation) and graphical summaries (case profile plots) were provided for each biomarker over time. In addition, plots of the relative change from baseline (%) were generated for each variable. There was no evidence against normality for the distributions of each biomarker at each time point. As each response variable of interest (i.e. the set of six biomarkers) is a continuous variable, a linear mixed model was used to model the change over time. A random effect for each individual was incorporated in all models and the within-individual correlation over time was specified as unstructured. The time when the testing was recorded was modeled as a fixed effect, initially as a categorical variable in order to allow a comparison in the mean change at each time point and then as a continuous variable in order to compare the slopes for each biomarker over time. Relationships between biomarker variables were examined using Pearson correlations. All statistical analyses were carried out using R (version 3.1) and the nlme package. The significance level was set at alpha = 0.05. Model assumptions were visually assessed for each response at each time point using residual plots from the fitted model.

### Part 1: Analytical variation (CV_A_)

The analytical coefficient of variation (CV_A_), the intra-assay CV_A_ (%) were calculated for the FORT and FORD test using methodology of Fraser and Harris [18]. CV_A_ is calculated using the following formula:

$${\text{CV}}_{\text{A}}=\frac{\text{SD}}{\text{X}}\;\times \;100\;(\%)$$

Where X = mean and SD = standard deviation

### Part 2: Analytical and Biological Variation

The CV_A_ for RBC GSH, lutein, and α and γ-tocopherols were calculated using the formula above, and derived from duplicate samples. The within subject biological variation (CV_w_), between subject variation (CV_B_), CDV, and index of individuality (II) were calculated according to methods of Fraser and Harris [18,19]. A missing value was substituted by the mean value for that participant in the analysis.

CDV was calculated using the following formula:

$$\text{CDV}=2^{1/2}•\text{Z}•{\left({\text{CV}}_{\text{A}}{}^{2}+{\text{CV}}_{\text{W}}{}^{2}\right)}^{1/2}$$

Given duplicate samples were run on RBC GSH, lutein, and α and γ-tocopherols, the CDV was adjusted to the following, where by n_2_ refers to the number of analytical replicates (duplicates):

$$\text{CDV for duplicate analysis}=2^{1/2}•\text{Z}•{\left({\text{CV}}_{\text{A}}{}^{2}/{\text{n}}_{2}+{\text{CV}}_{\text{W}}{}^{2}\right)}^{1/2}$$

II was calculated using the following formula:

$$\text{II}=\;{\left({\text{CVa}}^{2}+{\text{CV}}_{\text{w}}{}^{2}\right)}^{1/2}/{\text{CV}}_{\text{B}}$$

## Results

### Repeatability of FFT (part 1)

The repeatability of the FORT and FORD assay was 3.9% and 3.7% respectively. The FORT and FORD results for the squad were 1.66 (0.36) mmol•L^-1^ and 1.76 (0.17) mmol•L^-1^ respectively.

### Analytical, BV, CDV and index of individuality for the FFT (part 2)

**Fig 1:**
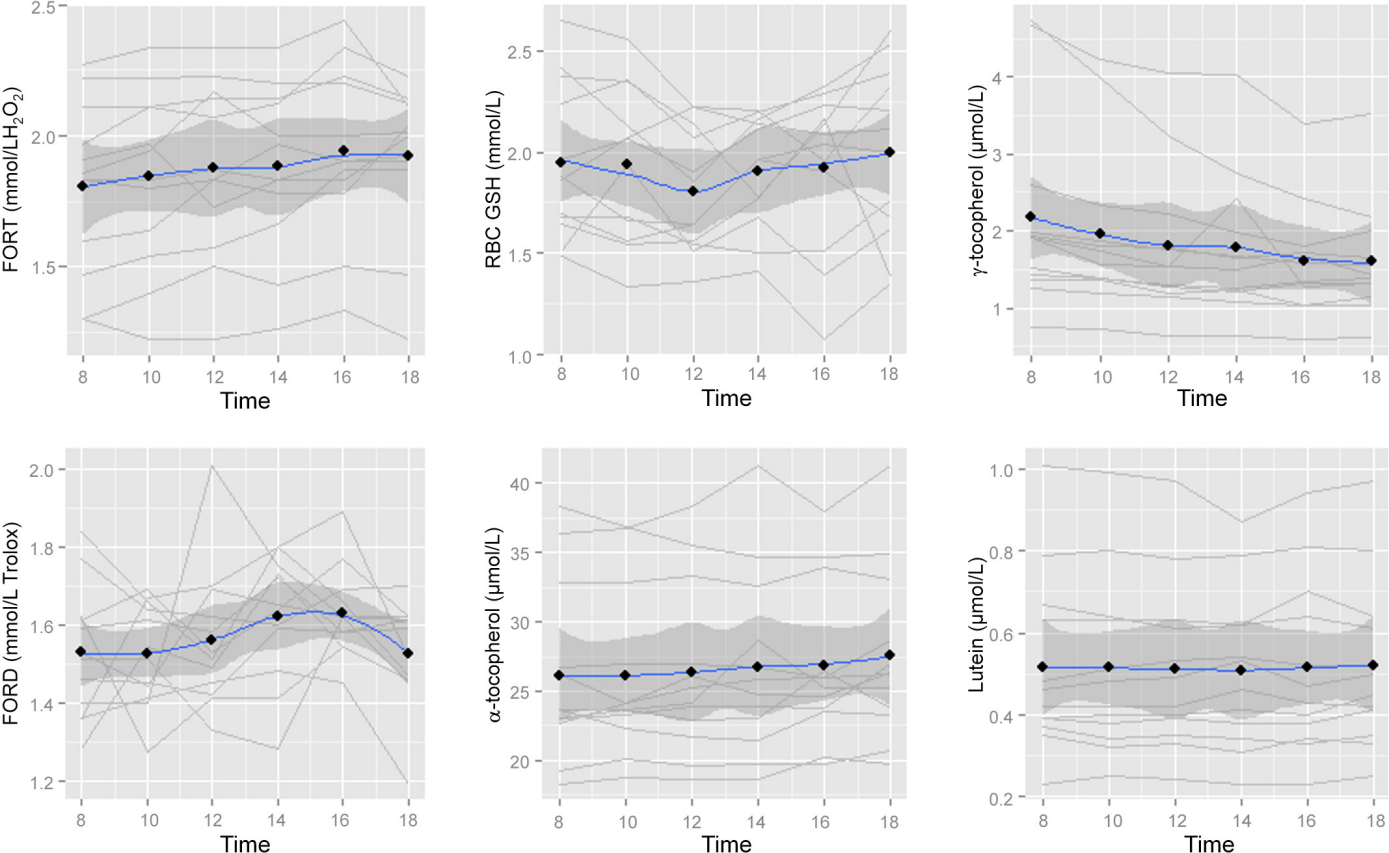
Case Profile plot of each biomarker over time with mean (black dot), smoothed trajectory (blue line) and 95% confidence interval displayed (dark shaded area).

A significant effect for time was observed for FORT (p<0.001), γ-tocopherol (p<0.001) and α-tocopherol (p = 0.002) over the 10-hours, indicating circadian variation (Fig 1).

**Fig 2:**
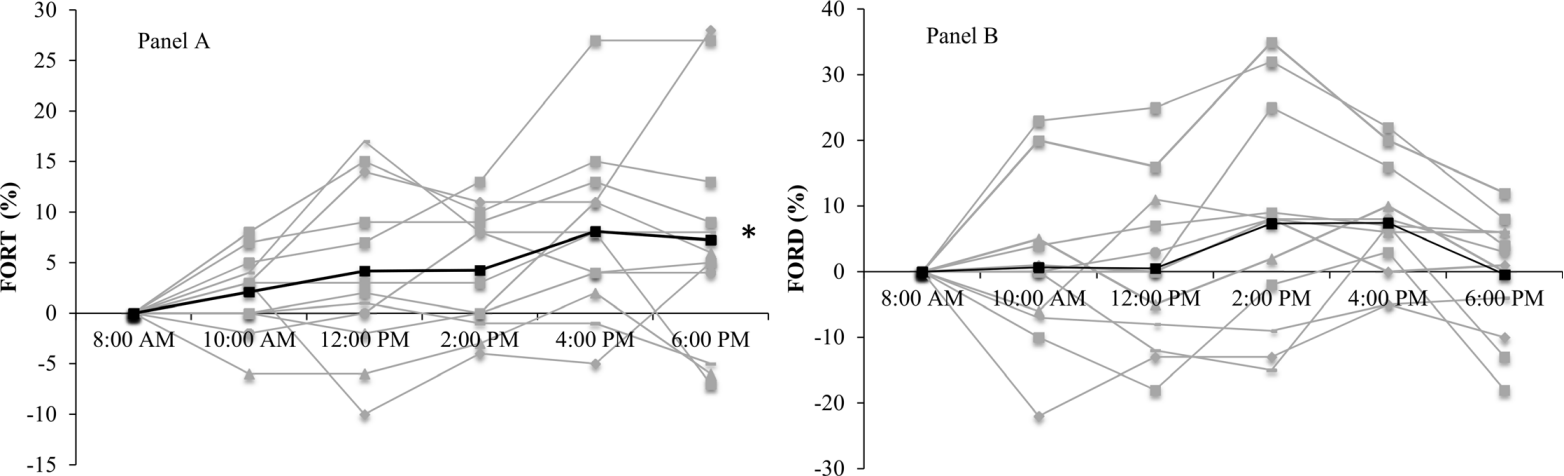
Relative changes (%) from baseline (time point 8:00 AM) for FORT (panel A) and FORD (panel B) over the 10-hours (n = 12). Dark black lines denote group average (mean) expressed as percentage change from baseline, with grey lines as individual responses. * Significant effect for FORT (panel A) over time (p<0.001).

**Fig 3:**
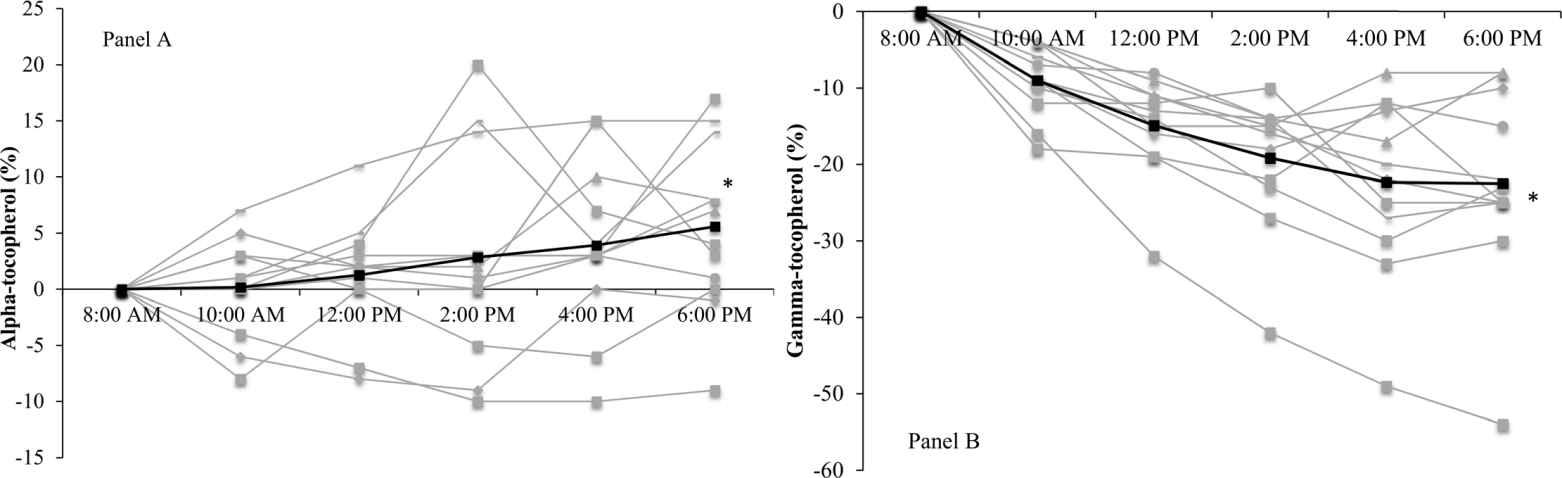
Relative changes (%) from baseline (time point 8:00 AM) for serum α-tocopherol (panel A) and γ-tocopherol (panel B) over the 10-hours (n = 12). Dark black lines denote group average (mean) expressed as percentage change from baseline, with grey lines as individual responses. * Significant effect for serum α-tocopherol (p< 0.001; panel A) and γ-tocopherol (p = 0.002; panel B) over time. *P*<0.05.

There was no effect for time for lutein (p = 0.60), RBC GSH (p = 0.52) and FORD (p = 0.26). Figs 2 and 3 show the temporal effect for FORT and FORD, α- and γ-tocopherols respectively, expressed as the relative change from baseline.

Table 1 summarises the mean (SD) for the FORT, FORD, RBC GSH, lutein and α and γ-tocopherols.

**Table 1:** Absolute mean ± SD concentrations for FORT, FORD, RBC GSH, lutein, and α and γ-tocopherols.

	8am	10am	12pm	2pm	4pm	6pm
FORT (mmol•L^-1^ H_2_O_2_)	1.81 (0.33)	1.84 (0.34)	1.88 (0.33)	1.88 (0.32)	1.94 (0.33)	1.92 (0.29)
FORD (mmol•L^-1^ Trolox)	1.53 (0.17)	1.52 (0.13)	1.56 (0.18)	1.62 (0.16)	1.63 (0.12)	1.52 (0.13)
γ–tocopherol (μmol•L^-1^)	2.17 (1.26)	1.97 (1.08)	1.81 (0.96)	1.79 (0.92)	1.60 (0.73)	1.60 (0.73)
α-tocopherol (μmol•L^-1^)	26.2 (6.4)	26.1 (6.1)	26.4 (6.2)	26.8 (6.6)	26.8 (5.7)	27.5 (6.2)
Lutein (μmol•L^-1^)	0.52 (0.22)	0.51 (0.22)	0.51 (0.21)	0.51 (0.19)	0.52 (0.21)	0.52 (0.21)
RBC GSH (mmol•L^-1^)	1.95 (0.39)	1.94 (0.38)	1.80 (0.30)	1.91 (0.28)	1.92 (0.39)	2.00 (0.43)

Table 2 summarises the analytical and biological variability, CDV and index of individuality for FORT, FORD, RBC GSH, lutein and α and γ-tocopherols. The analytical variability (CV_A_ %) for all the biomarkers indicates good precision for the assays (Table 2). The biomarker displaying the greatest analytical (6.8%), biological (12.5%), and between subject variability (51.4%), and CDV (37%) was γ-tocopherol.

**Table 2:** Analytical and biological variation, critical difference values and index of individuality for FORT, FORD, RBC GSH, lutein, and α and γ-tocopherols.

Biomarker	CV_A_ %	CV_W_ %	CV_B_ %	II	CDV %
FORT	3.9	5.0	17.3	0.29	17.4
FORD	3.7	7.5	9.6	0.78	23.8
γ-tocopherol	6.8	12.5	51.4	0.24	37.0
α-tocopherol	3.3	4.5	23.4	0.19	14.0
Lutein	3.5	3.9	40.8	0.10	12.8
RBC GSH	2.4	9.6	18.9	0.51	26.9

CV_A_ % = analytical variation, CV_W_ % = within subject, CV_B_ % = between subject variation, II = index of individuality, CDV % = critical difference value

For the redox biomarker FORT, moderate to weak relationships were observed for FORT and RBC GSH (r = -0.41; p < 0.001), α-tocopherol (r = 0.47; p < 0.001) and lutein (r = -0.24; p = 0.04) respectively. For plasma FORD, a strong relationship with RBC GSH was observed for AM measures only (p = 0.001; r = 0.62). For biomarkers of nutritional status; strong to moderate relationships were observed for α-tocopherol and γ-tocopherol (r = 0.78; p < 0.001); α-tocopherol and lutein (r = 0.37; p = 0.001); γ-tocopherol and lutein (r = 0.41; p < 0.001); and a weak correlation with RBC GSH and γ-tocopherol (r = 0.23; p = 0.04).

## Discussion

We present new information on the BV and accompanying CDV and index of individuality (II) for the OS and nutritional biomarkers FORT, FORD, RBC GSH, lutein and γ-tocopherol. Furthermore, we provide evidence of a significant circadian effect for FORT, and serum α and γ-tocopherol; no effect was observed for the antioxidants lutein, FORD or RBC GSH. A circadian effect for FORT and γ-tocopherol represents a finding not reported elsewhere. The repeatability (CV_A_) of the POC test for both the FORT (3.9%) and the FORD assay (3.7%), are comparable with reported laboratory measures of OS and are of sufficient analytical precision to be used clinically. Indeed, the CV_A_ for FORT shows better precision than that reported for malondialdehyde (MDA) (6.2%), lipid hydroperoxides (4.6%) plasma isoprostanes (4.5%), and protein carbonyls (11.9%) in BV OS research [9,20,26]. The CDV values vary between biomarkers, with large relative changes required for RBC GSH (>27%), FORD (24%) and γ-tocopherol (37%) before physiological significance could be confidently stated. For all OS and nutritional biomarkers reported here, the II indicates that reference ranges are of limited use in assessing meaningful changes in serial results in individuals. Interestingly, the participant with the largest relative increase in FORT (28% over the 10 hours), greater than the FORT RCV of 17% and thus deemed to be of physiological significance, had the lowest concentrations of the dietary antioxidants, γ-tocopherol, α-tocopherol, and lutein.

The increase in FORT observed over the course of the 10 hours might be explained by an increased mobilization of free fatty acids (FFA’s) into the circulation to provide energy in the absence of feeding (12–24 hour fast). Indeed, a positive correlation was observed between FORT and α-tocopherol, representative of increasing mobilisation of plasma α–tocopherol from adipose tissue in response to fasting, which is consistent with the observations of others [27]. Or, the increase in FORT may reflect a circadian effect for OS which is evident independent of feeding and has been reported by others for MDA, isoprostanes, and DNA oxidation [28]. In contrast to our findings, Davison *et al*. [9] observed no evidence for a circadian effect for MDA and lipid hydroperoxides over eight-hours in ten healthy fasted participants. The lack of an effect observed by Davison *et al*. [9], may result from the lower number of participants, the reduced period of observation, differences in oxidation by products measured and the greater analytical variability in the measures.

Given a circadian effect for biomarkers of OS and inflammatory cytokines (IL-6, TNF-α) and the antioxidant pineal hormone melatonin [29], the use of FFT in conjunction with the CDV may provide a means of monitoring the impact of significant disruptions in the circadian sleep-wake rhythm. Professional and Olympic athletes frequently undertake short- and long-haul flights and identifying athletes displaying disruption to their sleep-wake cycle (physiological relevant increases OS and inflammation using CDV) would aid athlete management. Increases in pro-inflammatory cytokines are observed after mild sleep deprivation [30], with FORT reported to correlate with inflammation (e.g. correlating with increases in high sensitivity c-reactive protein) [31].

Proposed theoretical mechanisms for the observed elevations in FORT (OS) are: 1) elevations in FFA oxidation as a result of increased FFA’s in keeping with a 24 hour fast and 2) coupled with reduced ATP demand-substrate oxidation (supine for 12 hours) and thus elevations in reducing equivalents (nicotinamide adenine dinucleotide; NADH_2_ and flavin adenine dinucleotide; FADH_2_) leading to increased mitochondrial superoxide and H_2_O_2_ formation and leak, ultimately elevating the basal levels of hydroperoxides. The generation of reducing equivalents being high during fatty acid metabolism, even at low physiological FFA concentrations [32] and the rate of mitochondrial H_2_O_2_ emission is increased when transitioning from carbohydrate to a high fat diet [33]. In addition, a strong relationship exists between plasma FFA’s and mitochondrial H_2_O_2_ production [34], and a 10-hour fast leads to persistent reductions in cytosolic GSH/GSSG [33]. We observed a significant inverse relationship between plasma FORT and RBC GSH concentrations.

To date three other studies have quantified the BV of OS indices and reported the CDV [9,20,26]. However, to our knowledge, no studies have reported BV or CDV data for FORT, FORD, RBC GSH, lutein and γ-tocopherol. The CDV we report in well trained participants for α-tocopherol of 14% is in close agreement with that of 13% reported by Davison *et al*. [9] in healthy males; using the same methodology. For FORT, we report an CDV of ~17%, which contrasts with that of Davison *et al*. [9] for measures specific to lipid peroxidation, with 28% for lipid hydroperoxides and 50% for MDA.

Our results demonstrate that laboratory reference ranges are not useful for the interpretation of OS biomarkers when applied to serial results in individuals, on the basis none of the biomarkers demonstrated an index of individuality greater than 0.8, with a low of 0.10 for lutein, and a high of 0.78 for the FORD assay. An index of greater than 1.4 indicates results can be evaluated usefully against reference ranges [18].

The strengths of this study are the level of methodological pre-analytical control applied to the participants. Such rigorous controls are a necessary feature of studies on BV to minimize sources of variability. A limitation of the present study is that participants were all male. Females were excluded given that oestrogen functions as an antioxidant [35]. In addition, the inclusion of validated biomarkers of lipid, protein or deoxyribonucleic acid (DNA) oxidative damage would have enabled deeper interrogation and understanding of the novel FORT assay in athletes e.g. F2-isoprostanes; advanced oxidation protein products; protein carbonyls or 8-Oxo-2'-deoxyguanosine; for a review in elite athletes see Lewis et al. [1]. In view of the fact plasma protein carbonyl concentrations have been shown to strongly reflect the redox status of tissue at rest and after exercise, namely skeletal muscle and heart [36]; the measurement of protein carbonyls would be recommended for future studies incorporating FORT measures.

We recognise that there are limitations with “total” antioxidant capacity assays (TAC) because such assays are not representative of the “total” antioxidant network in biological systems [37]. However, we chose to investigate the FFT, which includes a measure of plasma antioxidant capacity (FORD) because: 1) the FORD assay decreases in diseases of OS and is thus sensitive to depicting changes in OS; 2) uric acid is not a major component of the antioxidant activity of the assay; 3) measures of TAC have been shown to decrease with psychological stress, altitude, intense competition, training load and fatigued states [1,38]; 4) GSH is reported to contribute to the antioxidant activity of the assay, and thus explain a significant proportion of the variability in the assay 5) we believe the application of an OS point of care test has potential utility in elite sport and is worthy of scientific investigation. For plasma FORD, a significant strong relationship with RBC GSH was observed for a.m. only.

## Conclusions

We provide new information on the analytical, BV, II, CDV for biomarkers of OS and nutritional status, to be used for monitoring and assessing meaningful changes in serial results in individuals in relation to health, exercise and performance. We stress the importance of generating such data, given that for many biomarkers (notably redox measures), laboratory reference ranges have poor utility due to BV. In view of a circadian effect for FORT, α and γ-tocopherol, consideration should be given to the time of day for sampling. Finally, the FFT redox test may be applied in sports and clinical practice and in the field (e.g. training camps) for the assessment of OS, with the critical difference values published here used to enhance interpretation of meaningful changes in OS in individuals.
